# The Whole Genome Assembly and Comparative Genomic Research of* Thellungiella parvula* (Extremophile Crucifer) Mitochondrion

**DOI:** 10.1155/2016/5283628

**Published:** 2016-04-11

**Authors:** Xuelin Wang, Changwei Bi, Yiqing Xu, Suyun Wei, Xiaogang Dai, Tongming Yin, Ning Ye

**Affiliations:** ^1^College of Information Science and Technology, Nanjing Forestry University, No. 159, Longpan Road, Xuanwu District, Nanjing, Jiangsu 210037, China; ^2^School of Computer Science and Engineering, Southeast University, Nanjing, Jiangsu, China; ^3^College of Forest Resources and Environment, Nanjing Forestry University, No. 159, Longpan Road, Xuanwu District, Nanjing, Jiangsu 210037, China

## Abstract

The complete nucleotide sequences of the mitochondrial (mt) genome of an extremophile species* Thellungiella parvula *(*T. parvula*) have been determined with the lengths of 255,773 bp.* T. parvula *mt genome is a circular sequence and contains 32 protein-coding genes, 19 tRNA genes, and three ribosomal RNA genes with a 11.5% coding sequence. The base composition of 27.5% A, 27.5% T, 22.7% C, and 22.3% G in descending order shows a slight bias of 55% AT. Fifty-three repeats were identified in the mitochondrial genome of* T. parvula*, including 24 direct repeats, 28 tandem repeats (TRs), and one palindromic repeat. Furthermore, a total of 199 perfect microsatellites have been mined with a high A/T content (83.1%) through simple sequence repeat (SSR) analysis and they were distributed unevenly within this mitochondrial genome. We also analyzed other plant mitochondrial genomes' evolution in general, providing clues for the understanding of the evolution of organelles genomes in plants. Comparing with other Brassicaceae species,* T. parvula *is related to* Arabidopsis thaliana *whose characters of low temperature resistance have been well documented. This study will provide important genetic tools for other Brassicaceae species research and improve yields of economically important plants.

## 1. Introduction

Plant mitochondrial genome contains unique features, including large-scale sequence, frequent recombination, and merging foreign DNA [1]. Mitochondria are significant to life and play an important role in the cell. Mitochondria tackle oxidative phosphorylation very efficiently which is related to energy releasing in the electron transport and each molecule of glucose oxidized can produce about 30 molecules of ATP; however, only two molecules of ATP are produced through nuclear-controlled glycolysis. Toxic reactive oxygen species, a byproduct produced by mitochondria through oxidative phosphorylation increasing with age, can damage proteins, DNA, lipids, and their production [2]. As a typically active organelle with regularly undergoing fission and fusion, mitochondria have been thought to play an important part in mitochondrial outer membrane permeabilization process [3]. Another important feature of mitochondrial genome is its maternal inheritance, which shows its difference with nuclear DNA [2]. In plants, nuclear genes carry out relative copies of molecules of recombination-derived subgenomic DNA from mitochondria [4]. Additionally, a large set of mitochondria ribosomal proteins moves rapidly to the nucleus in certain angiosperm groups [5]. Although the evolution of plant mitochondrial genome sequence is slow, its structural evolution is swift [6]. The reported* Arabidopsis thaliana* mitochondrial genome sequence gives a more comprehensive and detailed view of internal duplication and foreign DNA uptake has influenced the size, structure, and evolutionary potential. Evolutionary surprises about plant mitochondrial genomes will continue to be brought out. However, within the high rate of mutation, only a very small part of plant mitochondrial genomes can be assembled.

The Brassicaceae family contains many species that are able to grow well in extreme conditions. Their stress tolerance mechanisms have been found in these species which are able to survive in multiple abiotic stresses environments including freezing temperature and high salinity. The abiotic stresses are disadvantageous to improve the yield of plants, so in order to improve productivity of economically important plants such as winter rape (*Brassica napus*), mustard (*Brassica juncea*), cabbage* (Brassica oleracea*), and radish (*Raphanus sativus*), it is crucial to study how crucifers withstand natural stress.* T. parvula* is a species in the Brassicaceae (subclade Eutremeae), and its whole genome has been reported (GenBank ID: AFAN00000000.1) and is similar to* Arabidopsis thaliana*. So far,* Arabidopsis thaliana*'s low-temperature-resistant ability has been well documented, which is also another advantage to study native crucifers [7].* Thellungiella parvula* mitochondrion plays significant roles in ATP synthesis, metabolism, and stress response. Because of its efficient mobilization of resources in poor or degraded soils,* T. parvula* perform better not only under salt conditions, but also in cold and freezing circumstance.* T. parvula* can live in the environment of 5°C, which means its temperature-sensitive processes such as seed germination and the production of pollen and seeds were resistant to cold temperatures. Moreover, the land plant shows dramatically different flowering phenotypes while shifting to cold environment [7]. It is expected that the research of the* T. parvula* mitochondrial complete genome sequence would serve as a reference and a useful genetic resource to the future research.

## 2. Results

### 2.1. *T. parvula* Mitochondrial Genome Content and Organization

*Thellungiella parvula* mitochondrial genome was assembled from the whole genome sequence using Roche-454 Sequencing technologies. The circular mitochondrial genome of* T. parvula* is 255,773 bp in size (GenBank accession number KT988071), with the overall base composition of 27.5% A, 27.5% T, 22.7% C, and 22.3% G in descending order and A + T content of 55.0% (Table 1). The overall GC content of the* T. parvula* mitogenome is 45%, which is similar to* Arabidopsis thaliana*, the first published mitochondrial genome sequence of the Brassicaceae family, originally from NCBI (NC_001284).

**Figure 1:**
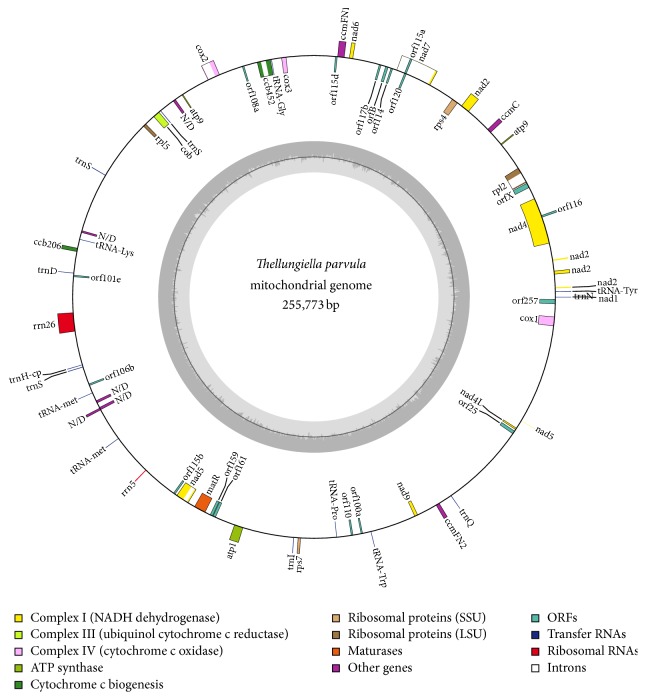
Circular gene map of the* Thellungiella parvula* mitochondrial genome. The* T. parvula* mitogenome consists of 54 genes, including 3 ribosomal RNA (rRNA) genes (5S rRNA, 26S rRNA, and rrnS), 19 transfer RNA (tRNA) genes, and 32 protein-coding genes.

Sequencing information such as uniquely aligned reads and average read depth of gene was shown in Table 6. Among all the 25 contigs, the longest is 73000 bp in length; its length value and N50 value are relatively large, suggesting that these sequencing data will be accurate and useful to assemble. Also, the encoding length of the largest contig is 51610 bp which accounts for 71%. Mapping these contigs to the completed mitochondrial genome sequence results of* Arabidopsis thaliana* showed that 20 of the contigs were assembled only once, while the other 5 contigs were linked twice or three times. Some certain sequences also have multiple copies in the mitogenome by analyzing these sequence results. Contig 1 was used at the beginning and we linked other contigs sequentially according to the connecting map (Figure 1). Finally, the* T. parvula* single master circle of mitochondrial genome covering the great mass of the contigs was generated with a complete length of 255773 bp.

Using web-based tool Public MITOFY Analysis [5] and tRNA scan-SE, 54 genes were identified, including 3 ribosomal RNA (rRNA) genes (5S rRNA, 26S rRNA, and rrnS), 19 transfer RNA (tRNA) genes, and 32 protein-coding genes (PCGs) (Table 2) (Figure 1). In addition, the positions of these genes in the* T. parvula* mitochondrial genome are shown in Table 1. Among genes of known function,* trnE-TTC* has two copies. By comparison with other* Arabidopsis thaliana*, the mitochondrial genome of* T. parvula* has the same types of function genes as crucifers.

The percentage of genes of known function is 11.5% (Table 7), 32 protein-coding genes in this mitogenome contain a total of 29,118 bp, and 9,706 codons are coded by nucleotide. Analyzing all the 32 protein-coding genes, the* nad5* is the longest (2009 bp) and the shortest is the* nad3* (119 bp). In these codons, 157 (1.62%) encode cysteine and 1071 (11.06%) encode leucine, which are the least and the most prevalent amino acids separately. AUU and UGC are used frequently to encode leucine and cysteine, respectively. Furthermore, nine genes have introns: four genes (*rpl2*,* ccmFn1*,* ccmFc*, and* cox2*) have a single intron, two genes (*nad4, nad2*) have three introns, and three genes (*nad1*,* nad5*, and* nad7*) have four introns. The complete NADH dehydrogenase subunit 2 (*nad2*) in* T. parvula* mitochondrial genome is encoded by five exons. These exons are located in two distant locations of the mitochondrial genome. One genomic region encodes exons a and b; the other encodes exons c, d, and e. As a result, the trans-splicing reactions are required to link exons b and c [8, 9].

**Figure 2:**
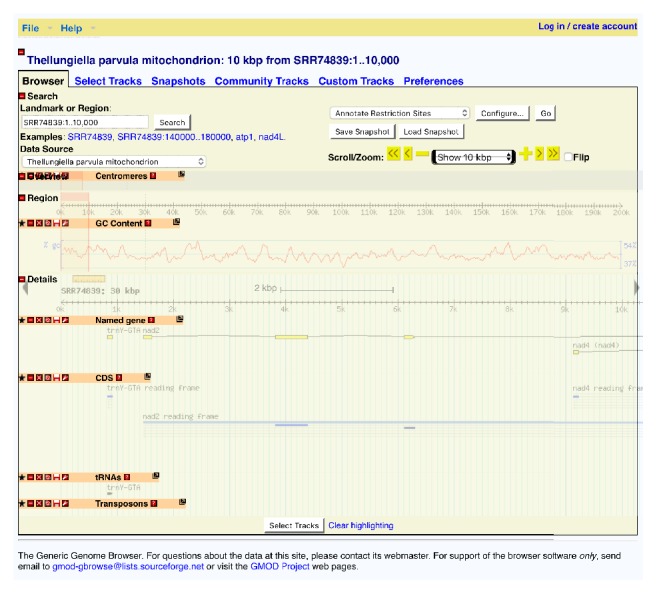
The GBrowse of the* Thellungiella parvula* mitogenome, showing the detailed location of mitogenome, GC content, CDs, tRNA, and other useful information.

The mitochondrial genome of* T. parvula *encoding tRNA and rRNA accounts for 0.6% and 2.01%, respectively. Noncoding sequences are present in a high rate (intergenic regions 76.132%, intron regions 8.457%, and exon regions 4.501%) (Table 7). Fourteen SNPs are identified: nine are synonymous and five are not synonymous (*cox3*,* cob*,* ccmB*,* atp9*, and* rpl5*). Additionally, we built a GBrowse (Figure 2) for the* T. parvula *mitogenome (http://bio.njfu.edu.cn/cgi-bin/gb2/gbrowse/Thellungiella_parvula/).

### 2.2. Gene Organization of* T. parvula* Mitochondrial Genome

It has been observed that 19 of the conserved 32 protein-coding genes produce components of the electron transport chain and ATP synthase: nine subunits of complex I (*nad1, nad2, nad3, nad4, nad4L, nad5, nad6, nad7, *and* nad9*), one subunit of complex III (*cob*), four subunits of complex IV (*cox1-1, cox1-2, cox2, *and* cox3*), and five subunits of complex V (*atp1, atp4, atp6, atp8, *and* atp9*). Five additional proteins are involved in the biogenesis of cytochrome c (*ccmB, ccmC, ccmFN-1, ccmFn-2, *and* ccmFC*). Another seven genes encode ribosomal proteins (*rpl2, rpl5, rpl16, rps3, rps4, rps7, *and* rps12*). The remaining genes encode maturase (*matR*). What is more, the gene ccmFN* in T. parvula* mitochondrial genome is divided into two reading frames. The 19 tRNA genes, especially including three* trnS-GCTs*, are significantly sufficient to decode all codons. Out of 54 genes, fourteen tRNA genes (*tRNA* ^*Tyr*^ *, tRNA* ^*Ser*^ *, tRNA* ^*Lys*^ *, tRNA* ^*Asp*^ *, tRNA* ^*His*^ *, tRNA* ^*Glu*^ *, trnfM, tRNA* ^*Met*^ *, tRNA* ^*Ile*^ *, tRNA* ^*Cys*^ *, tRNA* ^*Leu*^ *, tRNA* ^*Trp*^ *, tRNA* ^*Gln*^, and* tRNA* ^*Asn*^) and seven protein-coding genes (*ccmC, Nad6, CcmFn-2, Cox2, ccmB, Atp1, *and* Rps7*) are encoded on the H-strand; others are encoded on the L-strand (Table 2) [10]. Three rRNA genes (*rrn5, rrn26, *and* rrnS*) are highly conserved between the two mitochondrial genomes of Brassicaceae.

### 2.3. Repeat Structure and Sequence Analysis

A repeat is defined as a pair of sequences which has over 90% similarity [1]. Overall, 53 repeats are identified in the mitochondrial genome of* T. parvula*, including 28 tandem repeats (TRs), 24 direct repeats, and one palindromic repeat.

An amount of 28 TRs is recognized in* T. parvula*, which are fewer than* A. thaliana* (48 TRs) (Table 3). Among these repeats, the size of these tandem repeats is 6–69 bp. There are four long TRs (>100 bp) (with copy numbers of 51, 51, 69, and 69 separately) located in the intergenic spacer regions of* nad3-trnK-TTT*,* nad5-nad1*,* cox1-1-trnN-cp,* and* cox1-1-trnN-cp* of the* T. parvula* mitochondrial genome (Table 3). Tandem repeated sequences are allocated unevenly; repeat-rich sequences are widely concentrated near the central and the end regions.

**Figure 3:**
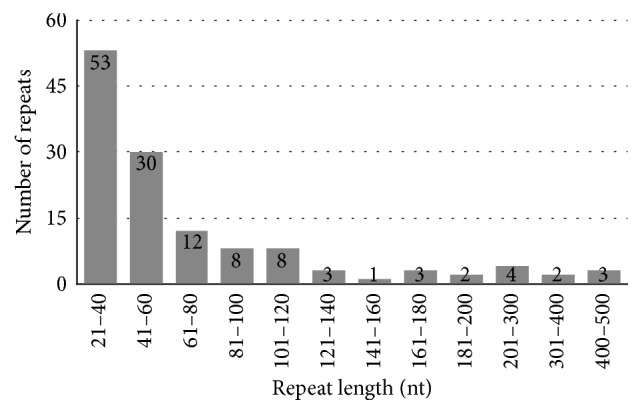
Frequency distribution of repeat lengths in the mitochondrial genome of* Thellungiella parvula*.

We used Blastn to align the* T. parvula* mitochondrial genome to locate and identify disperse repeats which has a minimal length of 100 bp and more than 90% identity between the two repeat copies, including forward repeats, inverted repeats, and palindromic repeats. As a result, the repeats in the* Thellungiella parvula* mitochondrial genome include sixteen forward repeats and eight inverted repeats (disperse repeats). We paid more attention to the 24 large repeats because they are related to reversible genomic structural changes. The large repeat of* T. parvula *mitochondrial genome is 10,084 bp in size, accounting for 3.9% of the whole mitogenome. Sixteen forward repeats and eight inverted repeats were among 106 and 453 bp (Figure 3). Most of these repeats are located in the no-protein-coding area; however, two inverted repeats are in the protein-coding regions and rRNA gene, respectively. These two genes in mitogenome that participated in energy metabolism probably have important effects on* T. parvula*. What is more, the length of palindromic repeat is 32 bp, which is distributed in the intergenic spacer regions of* ccmFc* and* cox2*. The disperse repeats research will offer significant information for the future population phylogeny study.

Simple sequence repeats (SSRs), also known as microsatellites, are usually 1–6 bp in length per unit and are widely distributed over the mitochondrial genome [11]. With a higher degree of polymorphism, codominant microsatellites are used as molecular marker assisting breeding [12], population genetics [13], genetic linkage map construction, and gene mapping [14]. A total of 199 simple sequences repeats were detected in the* T. parvula* mitochondrial genome, of which 4 are pentanucleotides, 6 are trinucleotides, and 189 are others. These SSRs have a high A/T content (83.1%) and the 3 dinucleotides are composed of AT (Table 4). The higher A/T content in SSRs of mitochondrial genome also contributes to a slight bias of 55% AT in the* T. parvula* mitogenome. Moreover, it is clear that SSRs distributed in the protein-coding areas are much fewer than noncoding regions, which account for 9.06% and 90.94%, respectively. Additionally, 32.01% of SSRs exist in the introns and 58.93% of SSRs exist in the intergenic spacer regions. However, SSRs account for only 0.1% of the whole protein-coding areas, which may further indicate an uneven distribution of SRRs within the mitochondrial genome of* T. parvula.*

### 2.4. Comparison with Other Brassicaceae Species

*T. parvula* completed mitochondrial genome sequence provides a unique view of mitochondrial genome structure, organization, and gene complement. Comparing with other Brassicaceae species, we conclude that the* T. parvula* has a similar mitochondrial sequence organization to that of most species, such as* Arabidopsis thaliana* (GenBank ID: Y08501.2) which is unquestionably a kind of stress-sensitive crucifers, so it is meaningful to compare its mitogenome with that of* Arabidopsis thaliana*. From the web-based tool Public MITOFY Analysis, the* T. parvula *mitochondrial genome has 5 ATPase and 9 NADH which their numbers are equal to* A. thaliana *mitochondrial genome, but ribosomal proteins are slightly higher in* T. parvula *[4] than that in* A. thaliana *[3, 5]. Also, it may reflect the different environment pressures and habitats between the species adapted. Ribosomal proteins S1 (*rps1*),* rps10,* and* rps11* have been completely lost in both species of the mitochondrial genome, which may indicate that genes transfer to nuclear compartment. In general, the base content of the* A. thaliana* mitochondrial genome is as follows: A (27.9%), T (27.3%), C (22.6%), and G (22.2%), which demonstrate a slight bias of A + T (55.2%) rich feature similar to that in* T. parvula* (Table 5). Except for* mttB* and* nad1* genes employing the ACG start codon, most of mitochondrial protein-coding genes of* A. thaliana* initiate with ATG; the usage of the stop one is rather complete or incomplete [15]. Four types of stop codons are used by the coding genes: TAA (*atp4, atp6, ccmB, ccmC, ccmFc, cox1, nad1, nad2, nad3, nad4L, nad9, rpl5, rpl6, rps4, rps7, *and* rps14*), TAG (*atp1, matR, sdh4, nad7, *and* rpl2*), TGA (*atp8, atp9, ccmFn, cob, cox2, cox3, nad4, nad6, *and* rps12*), and incomplete stop codons T- for* rps2, rps19*. Approximately, a total of 1098 and 646 tandem repeats were found in the* A. thaliana *and* T. parvula* genomes, respectively. In our initial analysis, this halophyte, with a genome about 30% smaller than that of* A. thaliana*, shows striking difference in gene complement. The differences are partly due to tandem duplications in* A. thaliana* of single copy genes in* T. parvula* and some genes amplification which may have known or assumed functions in stress defense responses [16].

### 2.5. Phylogenetic Analysis

**Figure 4:**
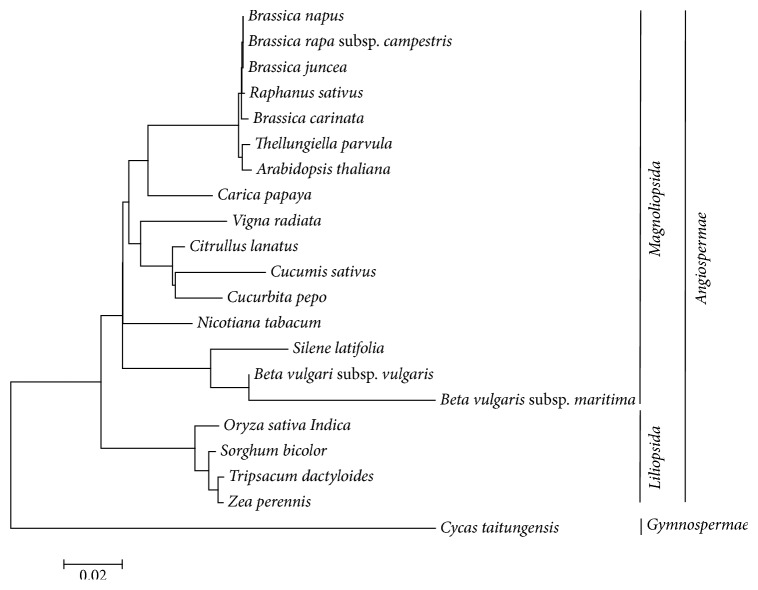
A phylogenetic context based on amino acid sequences of the fifteen protein-encoding genes (atp1, atp9, ccmB, cob, cox1, cox3, nad1, nad3, nad4, nad4L, nad6, nad7, nad9, rps3, and rps4) of twenty-one species (*Brassica napus, Brassica rapa *subsp.* campestris, Brassica juncea, Raphanus sativus, Brassica carinata, Thellungiella parvula, Arabidopsis thaliana, Carica papaya, Vigna radiata, Citrullus lanatus, Cucumis sativus, Cucurbita pepo, Nicotiana tabacum, Silene latifolia, Beta vulgaris *subsp.* vulgaris, Beta vulgaris *subsp.* maritima, Oryza sativa Indica, Sorghum bicolor, Tripsacum dactyloides, Zea perennis, *and* Cycas taitungensis*). These conserved genes were aligned with ClustalW and the phylogenetic tree was constructed using the Neighbor-Joining method in MEGA 6.

**Figure 5:**
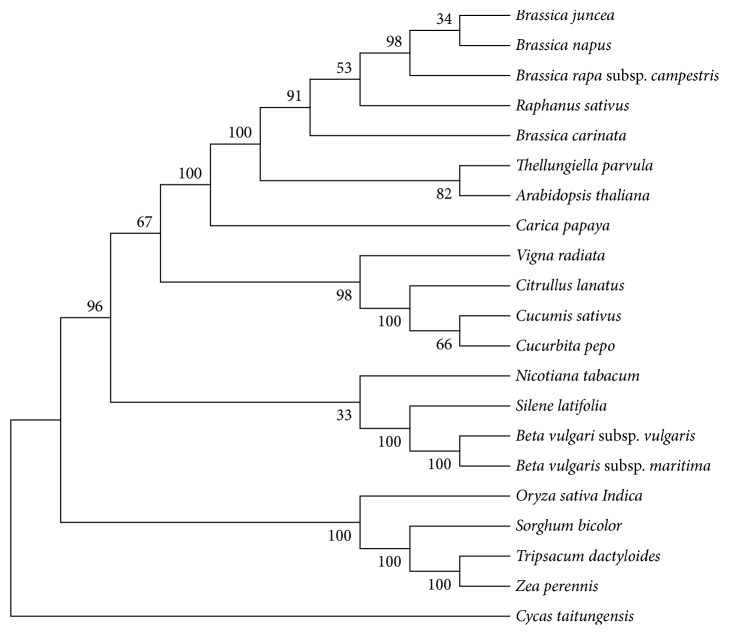
Maximum likelihood tree based on fifteen protein-encoding genes (atp1, atp9, ccmB, cob, cox1, cox3, nad1, nad3, nad4, nad4L, nad6, nad7, nad9, rps3, and rps4) of twenty-one species (*Brassica napus, Brassica rapa *subsp.* campestris, Brassica juncea, Raphanus sativus, Brassica carinata, Thellungiella parvula, Arabidopsis thaliana, Carica papaya, Vigna radiata, Citrullus lanatus, Cucumis sativus, Cucurbita pepo, Nicotiana tabacum, Silene latifolia, Beta vulgaris *subsp.* vulgaris, Beta vulgaris *subsp.* maritima, Oryza sativa Indica, Sorghum bicolor, Tripsacum dactyloides, Zea perennis, *and* Cycas taitungensis*). Numbers on each node are bootstrap support values.* Cycas taitungensis* is set as an out-group.

A phylogenetic context was based on amino acid sequences of the 15 protein-encoding genes (*atp1, atp9, ccmB, cob, cox1, cox3, nad1, nad3, nad4, nad4L, nad6, nad7, nad9, rps3, *and* rps4*) of 21 species (*Brassica napus, Brassica rapa *subsp.* campestris, Brassica juncea, Raphanus sativus, Brassica carinata, Thellungiella parvula, Arabidopsis thaliana, Carica papaya, Vigna radiata, Citrullus lanatus, Cucumis sativus, Cucurbita pepo, Nicotiana tabacum, Silene latifolia, Beta vulgaris *subsp.* vulgaris, Beta vulgaris *subsp.* maritima, Oryza sativa Indica, Sorghum bicolor, Tripsacum dactyloides, Zea perennis, *and* Cycas taitungensis*) (Figure 4). We got these twenty complete genome sequences from the GenBank of NCBI Organelle Genome Resources database (http://www.ncbi.nlm.nih.gov/genome/organelle/). Among these species, 20 are angiosperm species and one is a gymnosperm plant (*Cycas taitungensis*) that was set as an out-group. Twenty angiosperm plants are divided into two major clades: monocotyledons and dicotyledons plants. Bootstrap (Figure 5) analysis shows that there are 12 out of 18 nodes with bootstrap values >90%, and 8 of these have a bootstrap value of 100%. Those strong lineages conclude their sister relationship to Caricaceae and Brassicaceae (100% bootstrap values). In the NJ tree,* T. parvula* is near to* Arabidopsis thaliana*; although the bootstrap value is only 82%, the results are consistent with previous molecular studies [16]. Through the phylogenetic analyzing, the mitochondrial genome of* T. parvula* is evolutionarily close to that of* Brassica napus*,* Brassica Rapa *subsp.* campestris*,* Brassica juncea*,* Raphanus sativus*, and* Brassica carinata*. Then, the mitogenomes of* Carica papaya* and* Zea perennis* belong to Caricaceae and Gramineae, separately.

In the plants evolution, the numbers of protein-coding genes in mitochondrial genomes tend to decrease [17]. As a representative of gymnosperms, the mitochondrial genome of* Cycas taitungensis* has conserved almost all genes inherited from the seed plants, which was mitochondrial genome progenitor [18]. In contrast,* Silene latifolia* has lost all of the ribosome coding genes except for* rpl5* and other genes like the* sdh* [19]. Succinate dehydrogenase genes are usually missing. Almost all the monocotyledons and dicotyledons plants have lost* rps11* gene. This loss in the mitochondrial genome probably occurred in the early stage of the divergence process between monocotyledons and dicotyledons. The protein-coding gene like* mttB* that encodes a transport membrane protein in* T. parvula*,* Brassica napus,* and* Brassica carinata *is an exceptional losing one. More specially, contrasting with* Cycas taitungensis*, other twenty plants like* Thellungiella parvula, Brassica carinata, Brassica napus,* and* Zea perennis* have lost* rpl10, sdh3,* and* sdh4* genes, which can be inferred as* rpl10, sdh3,* and* sdh4* gradually developed into pseudogenes during the evolution of angiosperms. The loss of* rps2* in sixteen dicotyledons plants (*Brassica carinata, Brassica juncea, Brassica napus, Brassica rapa *subsp.* campestris, Raphanus sativus, Thellungiella parvula, Arabidopsis thaliana, Carica papaya, Vigna radiata, Citrullus lanatus, Cucumis sativus*,* Cucurbita pepo, Nicotiana tabacum, Silene latifolia, Beta vulgari *subsp.* vulgaris*, and* Beta vulgaris *subsp.* maritima*) probably occurred before the formation of Magnoliopsida plants, since most ribosome protein genes are frequently absent in clades of angiosperm mitochondrial genomes, which can be considered unnecessary to some extent [19].

Some chloroplast tRNA genes through random intercellular transfers have been inserted to mitochondrial genomes [20]. Many evolutionarily original species still maintained ancient transfers to some degree; meanwhile, new transfers exist in individual genera or species. These chloroplast genome genes* trnH, trnM, and trnS* (GGA) are regularly found in the mitochondrial genomes of* C. taitungensis* and the nineteen species mitogenomes (*Brassica carinata, Brassica juncea, Brassica napus, Brassica rapa *subsp.* campestris, Raphanus sativus, Thellungiella parvula, Arabidopsis thaliana, Carica papaya, Vigna radiata, Citrullus lanatus, Cucumis sativus*,* Cucurbita pepo, Nicotiana tabacum, Silene latifolia, Beta vulgari *subsp.* vulgaris, Beta vulgaris *subsp.* Maritima, Oryza sativa Indica, Sorghum bicolor, *and* Zea perennis*). It may be considered that this gene transfer phenomenon has occurred before the formation of angiosperms. Through analyzing species mitochondrial genome sequence in the phylogenetic tree (Figure 4), many mitochondrial-like tRNA genes may be lost in various plants, but the* trnE, trnP, *and* trnR* genes exist stably in mitochondrial genomes of these seed plants. Additionally, according to the analyzing of mitogenome sequence and phylogenetic context (Figure 4), seed plants' mitochondrial genomes are inserted into* trnH* but disappeared in the mitogenome of* Tripsacum dactyloides*.

Comparing with the typical gymnosperm* Cycas taitungensis*, which has the largest numbers of mitochondrial-like genes, the* T. parvula* mitochondrial genome has lost 10 genes, including seven protein-coding genes (*rps1, rps10, rps11, rps13, rps14, rps19, *and* sdh3*) and three tRNA genes (*tRNA-Phe, tRNA-Arg, *and* tRNA-Val*) (Table 2). Similarly, these 10 genes, which are lost in* T. parvula*, cannot be found in its related species* Arabidopsis thaliana*. As a result, these genes are not essential in gene expression or they can be duplicated by other genes [21]. However, other lost mitochondrial genes may be considered as some functional losses [22]. Furthermore, this may imply that gene loss will be regarded as the mitogenome evolutionary compaction in seed plants [23].

## 3. Discussion

Plants mitochondrial genome sequences are increased rapidly with the availability of sequencing technology [24]. Crops, a group that includes putative keystone species [25], are chosen as an obvious target to satisfy the most urgent desire to study and improve agronomically important species [26]. These plants are expanding potential comparisons at the biochemical and physiological level in particular environments with other existing genomic and genetic models [27]. As a result,* T. parvula* is such a plant. On one hand, it resembles the prototypical model* Arabidopsis thaliana* in development and evolution [28]. On the other hand, it is a plant with halophytic characters and exceptionally high mobilization of extreme environment, including salinity, cold, freezing temperatures, and the ability to grow in degraded soils [29]. With the* T. parvula* mitochondrial genome sequence presented in this study and also with reference to whole genome sequence of* T. parvula* [16], we will enlarge the exploration of genome sequence that favored extremophile adaptations in further researches.

The reported Brassicaceae species including* T. parvula* and* Arabidopsis thaliana* are 255773 bp and 366924 bp in size, respectively, both of them with a little smaller mitochondrial genome among higher plants [6]. As expected, genes contents of these mitochondrial genomes and the completed sequences are highly conserved between* T. parvula* and* A. thaliana*. Large repeats numbers (>1 kb) are less in Brassicaceae (*Arabidopsis* and* Thellungiella*) than in other species [30]. The repeat sequence detected in the* T. parvula* mitochondrial genome could be used as molecular marker and in population genetics studies, genetic linkage map construction, and gene mapping. Phylogenetic analysis also proved the two clades of Angiospermae and the isolation of Magnoliopsida with Liliopsida. Moreover, it proved the place of* T. parvula* in crucifer. So far, it has also been reported that the mitochondrial genomes of these crucifers do not contain much nuclear sequence plastid genomes, which can be one of the reasons why Brassicaceae species mitochondrial genomes are small. It seems that pathways and functions related to stress could be different in evolutionarily stress-tolerant plants ofBrassicaceae [31]. As a result, the mitogenome of* T. parvula* will be a useful tool in exploring extreme condition tolerance and mechanisms of adaptive evolution. Additionally, within these differences, we expect to learn the unique lifestyle of* T. parvula* and its adaptation to essential ecological niche.

The accomplishment of mitochondrial genome sequencing of both model plant and important crops in succession enlarged the higher plant mitochondrial genome database. However, there are still untypical plant mitogenomes. For instance,* Citrullus melo *L. has a large mitochondrial genome [32]. The mitogenome of Geraniaceae has the higher rate of nucleic acid replacement compared with homologous species [33]. These untypical mitochondrial genomes can also provide clues to the higher plants mitochondrial genome evolution mechanism. How the unknown mitochondrial genome sequences have emerged remains unsolved. We can carry out a research using mutants of crucifers related to genome distribution and the integrity of genome structure on the problems above in the future.

## 4. Methods

### 4.1. Plant Material and DNA Extraction

The original data are from GenBank of NCBI Genome Resources database (http://www.ncbi.nlm.nih.gov/genome?term=Thellungiella+parvula%5Borgn%5D&cmd=DetailsSearch). In their research, 10-day-old seedlings of* T. parvula* isolated total DNA are applied. The seeds were obtained from a single plant. At the collection site, the soil bulk density was 1.225 g/cm^3^ with 32.4% salts by weight [16]. By using the Nucleon Phytopure Genomic DNA Extraction kit (GE Healthcare), genomic DNA is prepared [16].

### 4.2. DNA Library Preparation and 454 Sequencing

The original data was constructed by both shotgun and paired-end libraries. To construct the two shotgun libraries, genomic DNA was nebulized to fragments of 500–800 bp randomly in size. Additional DNA was used to construct paired-end libraries with size spans of 3 kb (three libraries), 8 kb (two libraries), and 20 kb (two libraries). 454 Genome Sequencer FLX-Titanium was used to construct, amplify, and sequence all libraries according to the manufacturer's kits and protocols (454 Life Sciences) [16].

### 4.3. Mitogenome Sequence Assembly (2,155,540 Reads)

We expected to produce a gap-free, scaffold-level mitochondrial genome of the* T. parvula* through this research. Using Roche-454 FLX Titanium sequencing which has an advantage of read length, 2,155,540 reads with a total length of 6.2 G were obtained. The original sequence reads were a mixture of DNA with nucleus and other organelles. In order to assemble the mitogenome, we researched 359 plants mitochondrial genomes sequences from NCBI Organelle Genome Resources and found the common features; then, we used Blastn to isolate mitochondrion reads from the whole genome reads based on these completed reference mitogenomes. The sequences were assembled using Newbler 3.0 (454-Roche) with default parameters. During this process, we found 3801 contigs. Contigs with long length and high reads depth were separated with shorter ones [34]. The longest was 73000 bp in length. Contigs with reads depth between 50x and 100x may contain essential mitochondrial genes, so we filtered them out. To visualize the contigs connections, we used perl scripts and Newbler 3.0 generated file “454AllContigGraph.txt”. At the same time, according to reads depths of the contigs, false links and some wrong forks were removed manually [34]. Referring to the basis of the connecting map for the normal mitochondrion genome, we connected contigs to develop a circular mitochondrial genome and mapped them to* Arabidopsis thaliana* mitochondrial genome. After connecting 25 contigs, there were still some gaps. Contigs' gaps were filled up with a method like Ma et al.'s [35] study: first, we mapped mitochondrion reads onto both ends (3-60 bp) of the assembled contigs and then extended the contigs by joining the reads, which were partly overlapped (≥95% identical, *e* ≤ 1*E* − 30) with the contigs. Finally, a 255,773 bp nucleotide sequence was finished.

### 4.4. Genome Annotation

Genes were located using the web-based tool Public MITOFY Analysis [5], coupled with synonymous and nonsynonymous SNPs. tRNAscan-SE was used to identify the transfer RNA genes with default settings [36]. GC content was analyzed by perl script. The OGDraw version 1.1 was used to draw the circular mitochondrial genome map [37] (http://ogdraw.mpimp-golm.mpg.de/cgi-bin/ogdraw.pl).

### 4.5. Repeat Analysis

*T. parvula *mitochondrial genome tandem repeats were analyzed using Tandem Repeats Finder program [38] with default settings. Blastn alignments were used to identify and locate disperse repeats including the forward and palindromic repeats with a minimal length of 100 bp and over 90% identity between the two copies. For the simple sequence repeats (SSR), we used MISA [39] with the size of one to six nucleotides and the thresholds of eight, four, four, three, three, and three, respectively. Additionally, all kinds of repeats that were detected from the above programs were confirmed manually to simplify the results.

### 4.6. Phylogenetic Tree

The evolutionary tree of twenty species (*Brassica napus* (NC_008285),* Brassica rapa *subsp.* campestris* (NC_016125),* Brassica juncea* (NC_016123),* Raphanus sativus* (NC_018551),* Brassica carinata* (NC_016120),* Arabidopsis thaliana* (NC_001284),* Carica papaya* (NC_012116),* Vigna radiata* (NC_015121),* Citrullus lanatus* (NC_014043),* Cucumis sativus* (NC_016005),* Cucurbita pepo* (NC_014050),* Nicotiana tabacum* (NC_006581),* Silene latifolia* (NC_014487),* Beta vulgaris *subsp.* vulgaris* (NC_002511),* Beta vulgaris *subsp.* maritima* (NC_015099),* Oryza sativa Indica* (NC_007886),* Sorghum bicolor* (NC_008360),* Tripsacum dactyloides* (NC_008362),* Zea perennis* (NC_008331), and* Cycas taitungensis* (NC_010303)) has been inferred using the Neighbor-Joining method. Fifteen genes (*atp1, atp9, ccmB, cob, cox1, cox3, nad1, nad3, nad4, nad4L, nad6, nad7, nad9, rps3, *and* rps4*) were extracted by local perl scripts among these twenty-one plants. The evolutionary analyses including aligning multiple sequence by ClustalW, algorithm of Neighbor-Joining maximum likelihood (ML), Neighbor-Joining (NJ), and maximum parsimony (MP) used for tree building were conducted in MEGA 6.0 [19]. Numbers designate bootstrap (BP) index values (%) were calculated using 1000 replicates to estimate the support of the data for each internal branch of the phylogeny.

## Figures and Tables

**Table 1:** Overview of the *Thellungiella parvula* mitochondrial genome sequence.

Note	Size
A content	27.5%
T content	27.5%
C content	22.7%
G content	22.3%
A + T content	55%
Longest gene	2009 bp
Mean intron length	3740 bp

**Table 2:** Gene profile and organization of the *Thellungiella parvula* mitochondrial genome.

Gene/elements	Strand	Position	Size (bp)	Start codon	Stop codon	Amino acid	Anticodon	Read depth
tRNA^Tyr^	H	832–914	83	—	—		GTA	34.8
Nad2	—	1458–37186	1467	ATG	TAA	493	—	38.025
Nad4	—	9133–17081	1488	ATG	TGA	493	—	34.8
Rpl2	L	20135–22926	1050	ATG	TGA	349		34.8
Atp9	—	27712–87567	917	ATG	TGA	169		35.45
rrnS	H	158451–160299	1849	—	—	—	—	40.2
ccmC	H	30512–31255	744	ATG	TGA	247	—	34.8
Rps4	L	38009–39097	1089	ATG	TAA	362	—	33.9
Nad7	—	43566–43827	1185	ATG	TAG	394	—	33.9
Atp8	L	51211–51687	477	ATG	TGA	158	—	31.2
Nad6	H	57368–57985	618	ATG	TAA	205	—	37.9
CcmFn-1	—	58827–60048	1179	ATG	TAG	392	—	36.1
CcmFn-2	H	213390–214040	651	ACG	TAG	216	—	37.9
Nad1	—	63688–249532	978	ACG	TAA	325	—	37.68
Cox3	L	68658–69455	798	ATG	TGA	265	—	36.1
tRNA^Gly^	L	71129–71202	74	—	—	—	GCC	36.1
CcmFc	—	71321–73632	1359	ATG	TAA	452	—	36.1
Cox2	H	80299–82430	783	ATG	TAA	260	—	36.1
tRNA^Ser^	L	91957–92043	87	—	—	—	GCT	36.1
cob	L	92354–93535	1182	ATG	TGA	393	—	36.1
Rpl5	L	95345–95902	558	ATG	TAA	185	—	36.1
Rpl16	L	96020–96547	528	ACG	TAA	178	—	36.1
Rps3	L	96438–97964	1527	ACG	TAG	508	—	36.1
tRNA^Ser^	H	106353–106439	86	—	—	—	GCT	36.1
Rps12	L	116115–116492	378	ATG	TGA	125	—	36.1
Nad3	L	116538–116897	360	ATG	TAA	119	—	36.1
tRNA^Lys^	H	118042–118114	73	—	—	—	TTT	36.1
CcmB	H	119597–120217	621	ATG	TAA	206	—	36.1
tRNA^Asp^	H	123824–123897	74	—	—	—	GTC	36.1
Rrn26	H	130530–133703	3174	—	—	—	—	56.1
tRNA^His^	H	139455–139528	74	—	—	—	GTG	40.2
tRNA^Glu^	H	139709–139780	72	—	—	—	TTC	40.2
tRNA^Ser^	H	139828–139915	88	—	—	—	GCT	40.2
trnfM	H	144494–144567	74	—	—	—	CAT	40.2
tRNA^Met^	H	153356–153428	73	—	—	—	CAT	40.2
Rrn5	H	160405–160523	119	—	—	—	—	40.2
Nad5	—	167199–206340	2009	ATG	TAA	662	—	56.46
matR	L	170736–172709	1974	ATG	TAG	757	—	40.2
Atp1	H	178158–179681	1524	ATG	TAG	507	—	40.2
tRNA^Ile^	H	188375–188455	81	—	—	—	CAT	33.9
Rps7	H	189123–189569	447	ATG	TAA	148	—	33.9
tRNA^Cys^	H	195438–195508	71	—	—	—	GCA	33.9
tRNA^Pro^	L	195611–195685	75	—	—	—	TGG	33.9
tRNA^Glu^	H	195757–195828	72	—	—	—	TTC	33.9
tRNA^Leu^	H	198814–198898	85	—	—	—	CAA	33.9
tRNA^Trp^	H	201484–201557	74	—	—	—	CCA	35.5
Nad9	L	209345–209917	573	ATG	TAA	190	—	37.9
tRNA^Gln^	H	216365–216436	72	—	—	—	TTG	37.9
Atp6	L	223161–223604	444	ATG	TAA	147	—	37.9
Atp4	L	231218–231796	579	ATG	TAA	192	—	33.3
Nad4L	L	232069–232371	303	ATG	TAA	100	—	33.3
Cox1-1	L	251655–252425	771	ATG	TAA	256	—	35.8
Cox1-2	L	250843–251604	762	ATG	TAA	253	—	35.8
tRNA^Asn^	H	255697–255768	72	—	—	—	GTT	37.1

**Table 3:** Tandem repeats in the mitogenome of *Thellungiella parvula*.

Serial number	Start site	Stop site	Period size (nt)
1	2233	2313	31
2	5809	5857	22
3	20497	20553	28
4	54565	54599	18
5	60468	60526	25
6	60474	60531	25
7	84202	84231	14
8	102221	102251	9
9	112682	112709	14
10	113918	113993	26
11	114852	114903	27
12	117439	117537	51
13	123581	123667	36
14	123783	123820	20
15	183318	183381	33
16	208851	208888	19
17	211289	211358	29
18	216715	216788	27
19	222394	222429	18
20	226212	226255	18
21	234531	234630	51
22	235637	235709	27
23	236498	236549	16
24	236485	236549	32
25	237235	237287	19
26	253335	253492	69
27	253381	253554	69
28	254381	254405	12

**Table 4:** Distribution of SSRs in the mitogenome of *T. parvula*.

Type	SSR	Number of repeats	Start	End	Location
Monomer	T	9	763	771	—
	A	8	2074	2081	Nad2(intron)
	A	8	10932	10939	Nad4(intron)
	C	8	12096	12103	Nad4(intron)
	T	10	12253	12262	Nad4(intron)
	C	9	14435	14443	Nad4(intron)
	A	11	17525	17535	IGS(nad4, rpl2)
	T	10	19173	19182	IGS(nad4, rpl2)
	A	11	20944	20954	Rpl2(intron)
	A	8	25135	25142	IGS(rpl2, atp9)
	T	8	27558	27565	IGS(rpl2, atp9)
	C	11	35502	35512	Nad2(intron)
	C	10	39837	39846	IGS(rps4, nad7)
	A	10	43506	43515	IGS(nad4, rpl2)
	A	9	46361	46369	Nad7(intron)
	G	9	47843	47851	Nad7(intron)
	T	8	55038	55045	IGS(nad6, atp8)
	T	12	59173	59184	CcmFn1(intron)
	T	11	60629	60639	IGS(ccmFn1, nad1)
	T	8	63060	63067	IGS(ccmFn1, nad1)
	C	8	65079	65086	IGS(cox3, nad1)
	A	8	69152	69159	Cox3
	T	9	70078	70086	IGS(cox3, trnG-GCC)
	A	8	70989	70996	IGS(cox3, trnG-GCC)
	T	8	71627	71634	ccmFC
	T	8	73508	73515	ccmFC
	T	8	73782	73789	IGS(ccmFC, cox2)
	T	8	83553	83560	IGS(cox2, atp9)
	A	9	85876	85884	IGS(cox2, atp9)
	T	8	92114	92121	IGS(cob, trnS-GCT)
	A	9	94360	94368	IGS(cob, rpl5)
	T	8	96474	96481	Rpl16
	T	9	97650	97658	IGS(rps3, trnS-GCT)
	G	11	99474	99484	IGS(rps3, trnS-GCT)
	A	8	106875	106882	IGS(rps12, trnS-GCT)
	T	8	107254	107261	IGS(rps3, trnS-GCT)
	T	8	107957	107964	IGS(rps3, trnS-GCT)
	C	8	117336	117343	IGS(nad3, trnK-TTT)
	T	8	134167	134174	IGS(Rrn26, trnH-cp)
	T	8	137531	137538	IGS(Rrn26, trnH-cp)
	T	9	153501	153509	IGS(rrnS, trnM-cp)
	T	9	154495	154503	IGS(rrnS, trnM-cp)
	C	8	156682	156689	IGS(rrnS, trnM-cp)
	G	8	158985	158992	rrnS
	T	8	160651	160658	IGS(rrn5, nad5)
	G	9	166753	166761	IGS(rrn5, nad5)
	A	10	173435	173444	Nad5(intron)
	A	8	174660	174667	Nad5(intron)
	T	8	178106	178113	IGS(nad1, atp1)
	T	8	179843	179850	IGS(atp1, trnI-CAT)
	A	8	180779	180786	IGS(atp1, trnI-CAT)
	T	8	194226	194233	IGS(rps7, trnC-MT)
	A	11	195829	195839	IGS(trnE-TTC, trnL-CAA)
	T	9	210120	210128	IGS(nad9, ccmFn2)
	A	8	212337	212344	IGS(nad9, ccmFn2)
	G	10	231222	231231	Atp4
	T	8	233295	233302	IGS(nad4L, nad5)
	A	8	234008	234015	IGS(nad1, nad5)
	A	8	234745	234752	IGS(nad1, nad5)
	T	8	238579	238586	IGS(nad4L, nad5)
	G	9	245887	245895	IGS(nad4L, nad5)
	A	10	250105	250114	IGS(nad1, cox1)

Trimer	AAG	12	27383	27394	IGS(rpl2, atp9)
	AAC	12	78537	78548	IGS(ccmFC, cox2)
	AAG	12	80142	80153	IGS(ccmFC, cox2)
	ACA	12	86535	86546	IGS(atp9, cox2)
	AAT	12	87593	87604	IGS(atp9, trnS-GCT)
	CTT	21	134442	134462	IGS(rrn26, trnH-cp)

Pentamer	GTTCT	15	10356	10370	Nad4(intron)
	ATATG	15	52871	52885	IGS(atp8, nad6)
	TAATA	15	101932	101946	IGS(rps3, trnS-GCT)
	ATAGA	15	143566	143580	IGS(trnfM, trnS-GCT)

**Table 5:** Overview of the *Arabidopsis thaliana* mitochondrial genome sequence.

Note	Size
A content	27.9%
T content	27.3%
C content	22.6%
G content	22.2%
A + T content	55.2%
Longest gene	2019 bp

**Table 6:** Sequence information of the *Thellungiella parvula* mitochondrial genome.

Total raw reads (millions)	2.156
Uniquely aligned reads (millions)	0.075
Average read length (bp)	479
Duplication (%)	6.76
Sample input (*µ*g or ng)	50~100 *µ*g
Average read depth of gene	37
Average read depth of contigs	52.477

**Table 7:** The distribution of reads in the *Thellungiella parvula* mitochondrial genome.

Note	Percentage (%)
rRNA	2.010
tRNA	0.6
Intergenic	76.132
Intron	8.457
Exon	4.501
